# Supplementary figures and images for: Evaluation of immune and chemical precipitation methods for plasma exosome isolation
Source: PLoS One. 2020 Nov 24;15(11):e0242732. doi: 10.1371/journal.pone.0242732 (PMC7685508; doi:10.1371/journal.pone.0242732)

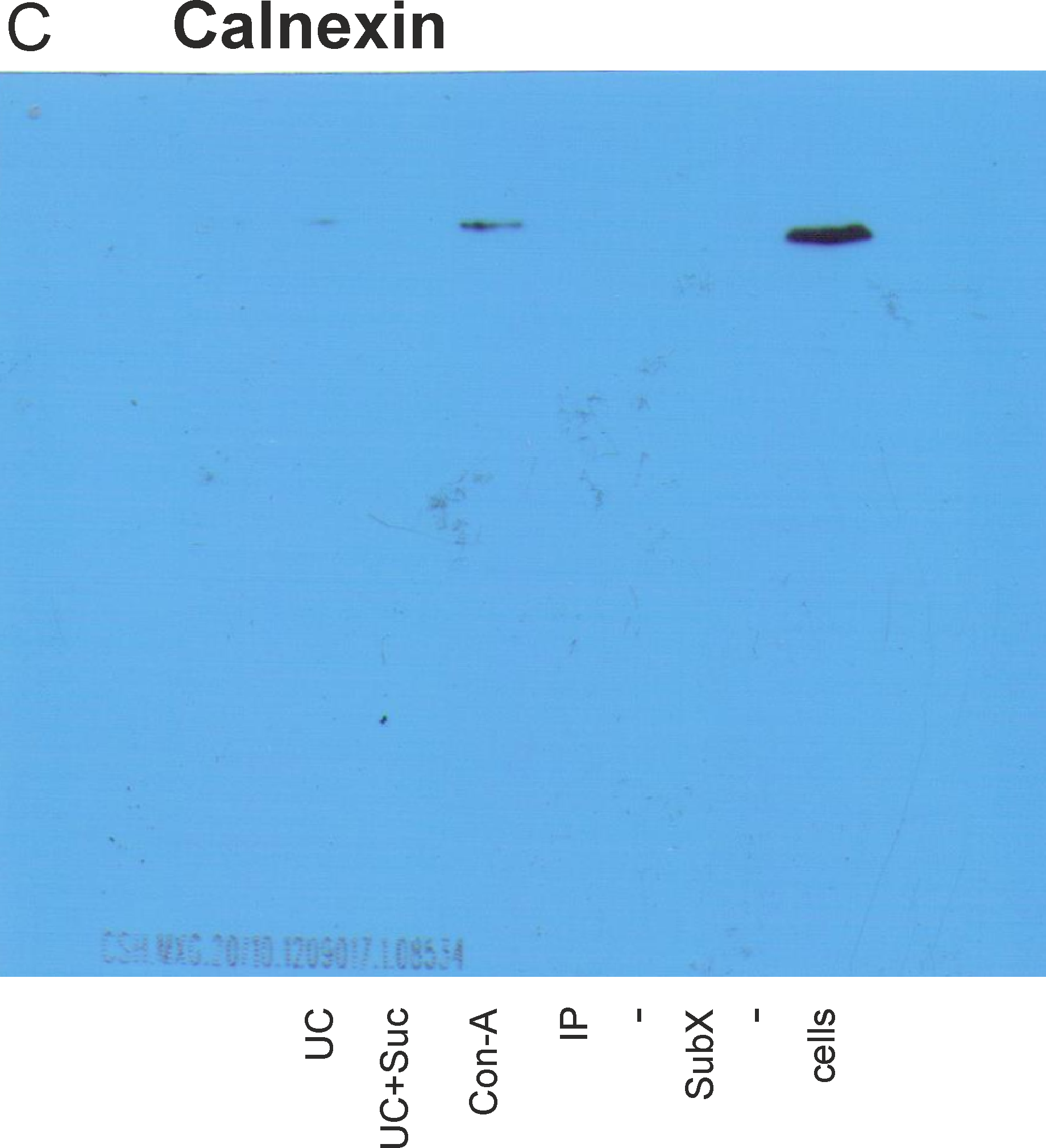

Supplement: S1 Fig — (TIF) [file pone.0242732.s001.tif]
